# Distinct phenolic, alkaloid and antioxidant profile in betel quids from four regions of Indonesia

**DOI:** 10.1038/s41598-020-73337-0

**Published:** 2020-10-01

**Authors:** Elizabeth Fitriana Sari, Grace Puspita Prayogo, Yit Tao Loo, Pangzhen Zhang, Michael John McCullough, Nicola Cirillo

**Affiliations:** 1 Melbourne Dental School, The University of Melbourne, 720 Swanston Street, Carlton, VIC 3053 Australia; 2 School of Agriculture and Food, Faculty of Veterinary and Agricultural Sciences, The University of Melbourne, Parkville, VIC 3052 Australia; 3 Faculty of Dentistry, Universitas Padjadjaran, Jl. Raya Sumedang KM 21, Jatinangor, 45363 Indonesia; 4 Dentistry and Oral Health, La Trobe Rural Health School, La Trobe University, Bendigo, VIC 3550 Australia

**Keywords:** Biochemistry, Cancer, Oncology

## Abstract

Betel quid (BQ) is a chewing mixed package that mainly contains areca nut (AN), betel leaf (Leaf) or betel stem inflorescence (SI), and slaked lime, and is consumed with or without tobacco BQ chewing is common in South East Asia and has been strongly associated with malignant and potentially malignant diseases of the oral cavity. Alkaloids such as arecoline are often accounted for the carcinogenic potential of BQ, however the chemical composition of BQ has not been studied in detail. In the current study, we investigated the total phenolic content (TPC), antioxidant activity (by mean of ferric reducing antioxidant power, FRAP), radical scavenging activity (DPPH test), polyphenolic profile and arecoline content in different components of BQ, namely AN, Leaf or SI, Husk, and blended BQ (BQ mix, containing AN, Leaf or SI and slaked lime). Samples were imported from 4 major regions of Indonesia, namely: Banda Aceh (BA), North Sumatra (NS), West Kalimantan (WK) and West Papua (WP). The highest TPC, FRAP, and DPPH values were detected in AN samples compared to other BQ components, while samples from WP region were of higher values compared to the other regions. High performance liquid chromatography—Mass Spectrometry (LC–MS) analysis showed that Husk contains the widest range of polyphenols, including hydroxybenzoic acids, hydroxycinnamic acids, flavanols, flavonols and stilbenes. Catechin and epicatechin were the main polyphenols detected in BQ, and they were present at the highest concentrations in WP–AN sample. Arecoline was detected in all AN and BQ mix samples and was significantly correlated with catechin and epicatechin, and significantly negatively correlated with *p*-hydroxybenzoic acid. Notably, arecoline concentration changed significantly when AN was blended in BQ mixtures. The current study is the first to extensively characterise the chemical composition of BQ and provides insight for a better understanding of the interactions of BQ alkaloids and phenolics in the development of oral submucous fibrosis and oral cancer.

## Introduction

The key risk factors of oral cancer include tobacco, alcohol, and betel quid (BQ) consumption^1,2^. In Western countries, the major risk factors of oral cancer are cigarette smoking and alcohol drinking, whereas in Asian countries such^3^ as Indonesia, the major risk factors are smoking and BQ chewing^3,4^. These same risk factors are also associated with the development of precursor lesions, overall known as oral potentially malignant disorders (OPMD). Indonesia has a poor cancer registry with limited studies conducted on the prevelance of oral cancer and OPMD, and their relation with BQ chewing^5^. Our recent epidemiological study, conducted in five different regions of Indonesia (Banda Aceh, West Java, Jakarta, West Kalimantan, and West Papua), showed that of 974 participants, 11.12% had oral potentially malignant disorders (OPMD)^6^, including submucous fibrosis. This previously underestimated prevalence of OPMD has major health implications as these conditions have a high potential to develop into oral cancer.

Areca nut (AN), the endosperm of the fruit of the Areca palm (*Areca catechu)*, is considered the key carcinogenic ingredient of BQ due to its high alkaloid content^7^. Four alkaloids, namely arecoline (most potent), arecaidine, guvacine, and guvacoline were reported to stimulate fibroblasts to produce collagen, thought to represents the main pathogenic mechanism of oral submucous fibrosis (OSMF), a potentially malignant condition most common in Indonesia^7,8^. The mutagenic effects of constituents and extracts of the AN have been studied extensively on cells^9^. However, AN is always consumed in a BQ package that contains additional components, most commonly betel leaf or betel stem inflorescence (SI) and slaked lime, which can affect the carcinogenic potential of AN extracts^10,11^. Therefore, it is important to investigate the chemical composition of all BQ ingredients.

Polyphenols are very important plant constituents and they exert antioxidant activity by inactivating lipid free radicals or preventing decomposition of hydroperoxides into free radicals^12^. However, polyphenols also possess pro-oxidant effect under certain conditions, such as high concentrations, high pH and the presence of redox-active transition metals^13–15^. The pro-oxidant activity of catechins at non-cytotoxic levels have been used as chemosensitizer for the treatment of cancer^13^. However, at high concentrations, certain polyphenols, such as catechins could exhibit cytotoxicity to normal cells^16–18^. Thus, moderate amounts of polyphenols could protect against diseases associated with oxidative stress such as cancer, coronary heart disease, inflammation via mechanisms like antioxidant activity and neutralisation/modulation of human/bacterial/viral proteins/enzymes^19,20^. Previously Chavan and Shinghal reported that catechin and epicatechin were identified from the *Mangalore* variety of AN^21^. Further study reported catechin as the most abundant polyphenols compound in AN from Malaysia^22^. Toxicity of AN are more likely contributed by polyphenols^23^. Several identified polyphenols in AN such as safrole, hydroxychavicol, and catechins have been proven as carcinogens^24,25^. Interestingly, catechin as the main flavanol present in BQ can also act as antimutagen and has been shown to prevent cell mutation in both oral cancer and leukoplakia cell lines by inhibiting the production of metalloproteases—a process potentially leading to a reduction of cell invasion and migration—and by inducing apoptosis and growth arrest^19^.

AN is consumed at different stages of ripeness, in raw form or after processing in many forms^26^. It has been reported that arecoline was the major alkaloid found in the immature nut^23^ and polyphenols were also found in higher amount in immature nut compared to mature nut^26^. A study by Senica et al. investigated the change of polyphenolic content in elder leaves (*Sambucus nigra* L.), flowers, and berries induced by different altitudes and locations^27^. The result showed that polyphenolic content was influenced by the altitude of plant’s growing site^27^.

Presently, there are limited studies investigating polyphenols and alkaloid composition of different components of BQ, and their potential correlation. The current study aims to fill this gap by exploring the polyphenols and alkaloid constitutes of areca nuts (AN) the endosperm of the fruit of *Areca catechu*, betel leaf of *Piper betle* (Leaf), betel stem inflorescence of *Piper betle* (SI), areca husk of *Areca catechu* (Husk) and fully packaged betel quid (BQ mix) originated from four key BQ consuming regions in Indonesia (BA, NS, WK, and WP). The location where BQ were imported can be seen in Fig S1. The current study also aims to establish correlations between different chemical compounds in BQ, which could inform further studies that investigate the interactions between alkaloids and phenolics in the development of OPMD and oral cancer.

## Methods and experimental design

### Chemicals and reagents

The reagents and solvents used for sample preparation and chemical assay were analytical reagent (AR) grades. AR grade methanol, sodium carbonate anhydrous, sodium hydroxide pellets and iron (II) sulfate heptahydrate were supplied by Chem-Supply Pty. Ltd. (Gillman SA, Australia). Hydrochloric acid (HCl) 36% grade AR, and iron (III) chloride anhydrous were purchased from Thermo Fisher Scientific (Scoresby VIC, Australia). Gallic acid, 2.0 N Folin-Ciocalteu’s phenol reagent (FCR), 2,4,6,-Tris(2-pyridyl)-s-triazine (TPTZ), 2,2-Diphenyl-1-picrylhydrazyl (DPPH), and (±)-6-Hydroxy-2,5,7,8-tetramethylchromane-2-carboxylic acid 97% (Trolox) were obtained from Sigma-Aldrich (Castle Hill NSW, Australia). Acetic acid glacial was supplied by VWR International Pty. Ltd. (Tingalpa QLD, Australia). The chemicals and standard compounds applied in HPLC analysis were in HPLC grades. Acetic acid 50% solution and standard compounds [gallic acid, 5-(hydroxymethyl)furfural, protocatechuic acid, caftaric acid, p-hydroxybenzoic acid, caffeic acid, syringic acid, p-coumaric acid, trans-sinapic acid, ferulic acid, procyanidin B1, procyanidin B2, catechin, epicatechin, epicatechin gallate, quercetin, quercetin 3-*O*-galactoside, quercetin 3-*O*-glucuronide, quercetin 3-*O*-rhamnoside, kaempferol, kaempferol 3-*O*-glucoside, resveratrol, polydatin were purchased from Sigma-Aldrich (Castle Hill NSW, Australia). Acetonitrile was obtained from Merck Pty. Ltd. (Bayswater, VIC, Australia). All water used was prepared by Milli-Q Gradient water purification system (Millipore Australia Pty. Ltd., North Ryde, NSW, Australia). The alkaloids analytical standard were arecoline hydrobromide 250 mg and guvacine hydrocloride 250 mg from Merck Pty.Ltd. (Castle Hill, NSW, Australia). Guvacoline hydrobromide 10 mg and arecidine hydrobromide 10 mg from Sapphire Bioscience Pty. Ltd (Redfern NSW 2016, Australia).

### Experimental design and sample preparation

**Figure 1:**
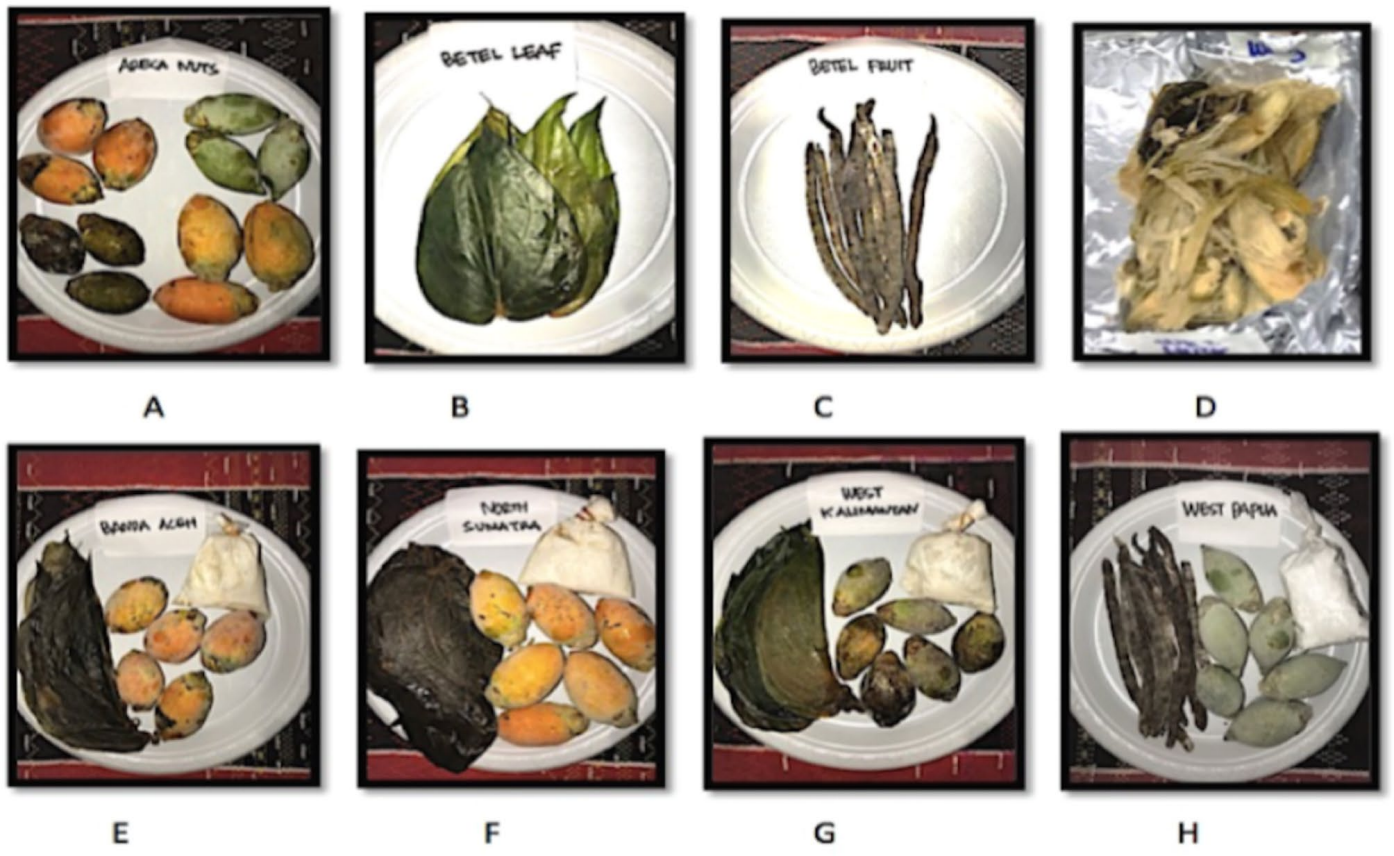
Different components of betel quid (BQ) samples from Indonesia. (**A**) Areca nuts, (**B**) Betel Leaf, (**C**) Betel Stem Inflorescence, (**D**) Husk, (**E**) BQ Mixture from BA (Banda Aceh), (**F**) BQ mixture from NS (North Sumatra), (**G**) BQ mixture from WK (West Kalimantan), (**H**) BQ mixture from WP (West Papua).

Raw BQ components were collected from four regions of Indonesia; Banda Aceh (BA), North Sumatra (NS), West Kalimantan (WK), and West Papua (WP). The four regions selected represented the west, middle, and east part of Indonesia. BQs were bought from local traditional market from each region. For BA, NS and WK regions, dried AN, Leaf, Husk, and BQ mix samples were analyzed, and the BQ mix contains dried AN, Leaf and lime at the ratio of 80.5:12.5:7 by weight^28^ (Fig. 1). For WP region, dried AN, SI, Husk, and BQ mix samples were analyzed, and different from other regions, the BQ from WP region contains, betel inflorescence stem (SI) that commonly named as flower, instead of betel leaf^26^. The outer cap colour differences of AN is a manifestation of the ripeness of AN, where the unripe AN looks green (shown by An from WP and WK regions), while the ripe AN looks yellow-orange colour^26^ shown by AN from BA and NS (Fig. 1).

Extraction of polyphenols from BQ samples were performed following published method with modifications^29^. Seeds and husks of AN were manually separated. All samples were freeze-dried for 72 h using FD3 Freeze Drier (Dynavac Engineering, Australia), and finely grinded using electronic grinder (Multigrinder II, model EM0405, Sunbeam, Auckland, New Zealand). 5 g of each sample powder was weighed into a 50 mL centrifuge tube prefilled with nitrogen gas (99.99% Grade 4.0, Coregas, Yennora NSW, Australia), added with 50 mL methanol as extraction solution. Extractions were carried out at 20 °C for 48 h under shaking condition at 150 rpm (Incubator shaker ZWYR-240, LABWIT scientific, Australia). After the extraction, the samples were centrifuged at 6500 rpm for 10 min at 20 °C. The supernatants were passed to a clean tube and evaporated at 40 °C to remove the solvent entirely using Hei-VAO Value rotary evaporator (Heidolp Instruments GmbH & Co.KG, Schwabach, Germany), then redissolved in 10 mL of LC grade methanol. The final extract was flushed with nitrogen gas and sealed with parafilm to avoid oxidation and storage at 4 °C in darkness until analysis. The extract stock solution was filtered using 30 mm × 0.45 µm nylon syringe filter (Thermo Fisher Scientific, VIC, Australia) to gain a clear solution before further analyses.

### Determination of total phenolic content (TPC)

The sample extract total phenolic content was examined using a modified Folin-Ciocalteu reagent (FCR) based on Singleton and Rossi (1965) method^30^. TPC measurement based on the spectrophotometric detection of chromogens established by the reaction between the phenols from the functional hydroxyl groups with the phosphomolybdic/phosphotungstic acid complexes reagents. A standard calibration curve was constructed using gallic acid at concentrations from 50 mg/L to 500 mg/L, with 50 mg/L increment. The total phenolic content of sample was expressed as gallic acid equivalents (GAE) [mg GAE/g sample dry mass (DM)]. The assay was conducted by adding 100 µL of diluted samples into clean tube containing 2.5 mL 0.2 N FCR (tenfold diluted 2.0 N FCR with Milli-Q water). Add 2 mL of saturated sodium carbonate solution (75 g/L) after 5 min. The blend solutions were vortexed and allowed to react in the dark room for 1 h. The absorbance was further quantified using Multiskan GO Microplate Spectrophotometer (Thermo Fisher Scientific Oy, Vantaa, Finland) at 765 nm. Technical duplicates were performed for each sample replicate.

### Determination of ferric reducing antioxidant power (FRAP)

The FRAP test of the sample extract was performed following the Benzie and Strain (1996) method^31^. FRAP assay uses antioxidants iron (III) as reductants in a redox-linked colorimetric method, employing an easily reduced oxidant system presenting stoichiometric excess. Furthermore, Iron (II) sulfate heptahydrate was used as the standard composite to produce the calibration curve of Fe^2+^ standard solution with the following concentrations: 5 µM to 45 µM Fe^2+^ standard solution at 5 µM increment. Results were presented as the Fe^2+^ equivalents (mM Fe^2+^ eq./g sample DM). All reagents were prepared fresh on the day of examination. 5 µL of diluted sample extract were added to 1.5 mL centrifuge tube pre-filled with 495 µL water and 1000 µL FRAP reagent. The solution was mixed completely with vortex before incubation process at 37 °C for 5 min. The absorbance of the solution was quantified at 593 nm using Multiskan GO Microplate Spectrophotometer (Thermo Fisher Scientific Oy, Vantaa, Finland). Technical duplicates were performed for each sample replicate.

### Determination of DPPH radical scavenging capacity

The DPPH analysis was performed based on Gadow et al. (1996) with modifications^32^. The DPPH 1,1-diphenil-2-picrylhydrazyl test measured the radical scavenging capacity of antioxidant by donating hydrogen to the DPPH radical. The standard compound using Trolox to construct the calibration curve. The Trolox concentration were presented as: 12.5 mg/L, 25 mg/L, 50 mg/L, 62.5 mg/L, 100 mg/L, 125 mg/L, and 250 mg/L. The result were expressed as Trolox equivalents (mg Trolox eq./g sample DM). 75 µL of diluted sample extract was added into 1425 µL of DPPH working solution in 1.5 mL centrifuge tube, vortexed and left in a darkness for 1 h at room temperature to enable reactions. The absorbance then examined at 515 nm using Multiskan GO Microplate Spectrophotometer (Thermo Fisher Scientific Oy, Vantaa, Finland). Technical duplicates were performed for each sample replicate.

### Characterisation of phenolic by HPLC (high-performance liquid chromatography) analysis

The HPLC phenolic characterization was performed by separation module Waters 2690 Alliance (Waters, Rydamere, NSW, Australia), paired with Waters 2998 Photodiode Array (PDA) detector following published protocols with modifications^21^.The separation was executed using a Synergi Hydro-RP 80A, LC Column (250 mm × 4.6 internal diameter, 4 µm particle size) (Phenomenex Inc., Lane Cove West, NSW, Australia). The segregation of phenolics was operated at 23 °C for column temperature and acquisition range of 200–600 nm with 1.25 scan/s (peak width = 0.2 min) of spectral acquisition rate was placed for the photodiode array detector.

### System I (phenolic acid, flavanols and stilbenes)

The mobile phase involved solvent A [acetic acid in Milli-Q water (2:98, v/v)] and solvent B [0.5% (v/v)]. A gradient program was used as the following: solvent B increased from 10 to 15% in the first 10 min followed by isocratic of solvent B at 15% for 5 min. Solvent B was increased from 15 to 25% for 10 min, then further rose from 25 to 35% for 30 min. Afterwards, solvent B increased from 35 to 55% within 30 min, then increased 55% to 100% for 1 min and kept at 100% for 5 min. After that decrease the solvent B from 100 to 10% in 4 min. The total run time was 70 min. The flow rate of the elution was maintained constant at 1.0 mL/min. All samples were injected by volume 10 µL. Synchronous detection wavelength was performed at 280 nm for hydroxybenzoic acids and flavanols and 320 nm for hydroxycinnamic acids and stilbenes.

### System II (flavonols)

System II used the similar composition of solvent A and solvent B as determined in System I (Phenolic acids), with a gradient program: solvent B rose from 10 to 24% in the initial 20 min, then increased to 30% in 20 min, and eventually improved from 30 to 55% for 30 min. Later, an increase of solvent B from 55 to 100% performed for 5 min, then kept at 100% for 5 min, finally decreased from 100 to 10% in 2 min. The total run time od elution was 82 min, and the flow rate was maintained constant at 1.0 mL/min. All samples were injected by volume 10 µL. Synchronous detection wavelength was performed at 370 nm for flavonols.

### System III (alkaloid)

The isocratic mobile phase composed of 50% acetonitrile and 50% of 0.01 M sodium hydrogenphosphate, adjusted at pH 7.8 by formic acid. The flow rate was 0.8 mL/min passing through the Synergy Hydro-RP 80 Å, (250 mm × 4.6 mm internal diameter, 4 µm particle size) (Phenomenex Inc., Lane Cove West, NSW, Australia). Total run time 10 min. The column temperature was 30 °C. The UV detector was set up at 216 nm^33^.

### LC-DAD-ESIMS analysis

LC–MS analysis of polyphenols was performed with Agilent 1260 Infinity II LC system (Agilent Technology, Santa Clara, United States)**,** coupled to Agilent ESI MSD (G6125B, Agilent Technology). Data acquisition and machine control were achieved using the Openlab Chemstation software. Negative ion mass spectra of the column elute were analysed in simultaneous sim/scan mode for polyphenols based on a published method^29^. The scan covered the range *m/z* 90–600, while the target sim ions used for individual compounds are listed in Table S1. Nitrogen was used both as drying gas at the flow of 11.0 L/min and as nebulizing gas at pressure of 55 psi. The nebulizer temperature was set at 350 °C. The LC elusion program used was the same as the System I, with DAD synchronous detection wavelength set at 280 nm, 320 nm and 370 nm.

### Statistical analysis

Statistical analysis was conducted using MS-Excel, Minitab 17 2016, MATLAB by Mathworks, XLStat, and R software (v3.5.2 The R foundation). One-way ANOVA with Odd Ratio test were executed at *p* ˂ 0.05. Group indicated with different letters showed statistically significant difference. The results were exhibited as the means ± standard deviation (SD) from four observations of individualistic replicates.

## Results

### Total phenolic content (TPC) and antioxidant activities in betel quid samples

The highest TPC was found in AN group from WP (216.71 mg GAE/g DM) followed by AN from BA (122.27 mg GAE/g DM), NS (111.72 mg GAE/g DM), and WK (108.84 mg GAE/g DM) respectively. The TPC value of WP-AN was almost twofold that of nut from other regions, indicating that WP-AN contains the most abundant phenolics among nut group (Table 1).

**Table 1:** Total phenolics content and antioxidant activities in AN, betel leaf/ betel stem inflorescence, BQ mixture and husk.

Samples	TPC mg GAE g-1 DM	FRAP mM Fe2 + eq./g sample DM	DPPH mg TE/g sample DM
BA-AN	122.27 ± 8.09***c***	62.59 ± 8.41***c***	277.43 ± 9.69***c***
NS-AN	111.72 ± 10.5cd	60.78 ± 3.83*c*	280.06 ± 2.21*c*
WK-AN	108.84 ± 8.81*d*	56.61 ± 7.34*c*	267.91 ± 19.26*c*
WP-AN	**216.71 ± 18.06a**	**107.77 ± 7.33a**	**422.53 ± 1.11a**
BA-Leaf	26.710 ± 1.94f.	18.24 ± 1.62*ef*	55.08 ± 5.13*fg*
NS-Leaf	19.91 ± 2.4f.	15.39 ± 1.5*ef*	42.67 ± 6.49*g*
WK-Leaf	63.93 ± 4.78*e*	31.99 ± 3.79*d*	111.28 ± 13.41*d*
WP-SI	59.00 ± 3.79*e*	21.61 ± 3.28*e*	71.73 ± 13.64*e*
BA-BQ mix	6.70 ± 0.69*g*	4.91 ± 0.43*h*	16.45 ± 3.19*hi*
NS-BQ mix	10.57 ± 3.36*g*	6.01 ± 1.18*g*	26.86 ± 8.72*h*
WK-BQ mix	27.69 ± 3.84f.	12.78 ± 1.78*fg*	62.52 ± 8.51*ef*
WP-BQ mix	155.09 ± 11.13*b*	94.60 ± 8.14*b*	303.34 ± 0.72*b*
BA-Husk	3.13 ± 0.26*g*	1.66 ± 0.06*h*	3.63 ± 0.64*i*
NS-Husk	5.14 ± 0.54*g*	2.76 ± 0.15*h*	9.62 ± 0.82*i*
WK-Husk	3.05 ± 0.24*g*	1.61 ± 0.03*h*	4.69 ± 0.48*i*
WP-Husk	5.36 ± 0.20*g*	2.45 ± 0.21*h*	8.29 ± 0.96*i*

Data are expressed as mean ± standard deviation of triplicate experiments (*n* = 3) with confident interval significantly (*P* < 0.05). Mean values in the same column followed by the same letter do not differ significantly. An *a* letter besides mean values refer to the highest value, and its value decreases following the descending letter in the same column.

*BA* = Banda Aceh, *NS* = North Sumatra, *WK* = West Kalimantan, *WP* = West Papua, *AN* = Areca Nut, *SI* = stem inflorescence *BQ Mix* = Areca nut + betel leaf/betel stem inflorescence stem + slaked lime, *TPC* = total phenolic content, *FRAP* = ferric reducing antioxidant power, *DPPH* = 1,1-diphenil-2-picrylhydrazyl.

The bold value is significantly higher compared to another mean in the same column.

The unbold value is significantly lower mean values in the same column.

There was a considerable reduction of TPC and antioxidant activities (FRAP and DPPH scavenging activities) when AN was in a BQ mixture with slaked lime (AN: betel leaf/stem: slaked lime = 80.5:12.5:7 by weight). In the betel leaf/stem group alone, WK-Leaf had the highest phenolics and antioxidant activities, while the least phenolics content was found in NS-Leaf.

### Polyphenolic content in betel quid components

Our study shows that phenolic acids were mostly detected in husk, leaf and SI samples, but traced in nuts and BQ mixtures. The most abundant phenolic acids detected were protocatechuic acid, *p*-hydroxybenzoic acid and syringic acid, while gallic acid, caffeic acid, p-coumaric acid, ferulic acid and sinapic acid were detected at low concentrations. The highest protocatechuic acid content was observed in NS-Husk followed by WK-Husk and BA-Husk respectively. The highest p-hydroxybenzoic acid was observed in WP-SI, followed by NS-Leaf, BA-Leaf, and WK-Leaf respectively. NS-Leaf has the highest concentrations of syringic acid (Table 2 and Fig S2).

**Table 2:** Polyphenols identified by LC–MS in BQ samples originated from different Indonesia regions (mg/g DM).

	BA-AN	BA-LEAF	BA-HUSK	BA-BQ MIX	NS-AN	NS-LEAF	NS-HUSK	NS-BQ MIX	WK-AN	WK-LEAF	WK-HUSK	WK-BQ MIX	WP-AN	WP-SI	WP-HUSK	WP-BQ MIX
**Phenolic acids**																
Gallic acid	ND	ND	ND	ND	ND	0.039 ± 0.06	13.42 ± 4.31	ND	ND	ND	ND	ND	ND	ND	ND	ND
5-(hydroxymethyl)furfural	ND	ND	ND	ND	ND	ND	ND	ND	ND	ND	ND	ND	ND	ND	ND	ND
Protocatechuic acid	ND	ND	0.25 ± 0.037	ND	ND	ND	6.99 ± 0.223	ND	ND	ND	0.40 ± 0.001	ND	ND	ND	ND	ND
p-hydroxybenzoic acid	ND	0.37 ± 0.082	0.114 ± 0.09	0.005 ± 0.001	ND	0.949 ± 0.75	ND	ND	ND	0.065 ± 0.014	0.43 ± 0.002	ND	ND	1.01 ± 0.02	0.33 ± 0.065	ND
Caffeic acid	ND	ND	0.004 ± 0.002	ND	ND	0.01 ± 0.002	0.006 ± 0.001	ND	ND	0.0037 ± 0.001	ND	ND	ND	ND	ND	ND
Caftaric acid	ND	ND	ND	ND	ND	ND	ND	ND	ND	ND	ND	ND	ND	ND	ND	ND
Syringic acid	ND	0.37 ± 0.015	0.324 ± 0.27	ND	ND	0.86 ± 0.04	0.28 ± 0.002	ND	ND	ND	0.02 ± 0.005	ND	ND	0.011 ± 0.006	0.094 ± 0.003	ND
p-coumaric acid	ND	ND	0.022 ± 0.002	ND	ND	ND	ND	ND	ND	0.004 ± 0.00	ND	ND	ND	ND	ND	ND
Ferulic acid	ND	ND	0.0005 ± 0.0	ND	ND	ND	ND	ND	ND	0.005 ± 0.002	0.015 ± 0.003	ND	ND	0.005 ± 0.001	0.005 ± 0.002	ND
Sinapic acid	ND	ND	0.074 ± 0.003	ND	ND	ND	ND	ND	ND	ND	ND	ND	0.015 ± 0.0005	ND	0.008 ± 0.003	0.015 ± 0.0004
**Anthoxanthins and stilbenes**																
Procyanidin B1	ND	ND	ND	ND	ND	ND	ND	ND	ND	ND	ND	ND	ND	ND	ND	ND
Catechin	9.699 ± 0.96	0.364 ± 0.02	0.276 ± 0.21	0.717 ± 0.102	8.51 ± 1.03	0.469 ± 0.12	0.379 ± 0.03	1.107 ± 0.22	2.335 ± 1.3	ND	0.059 ± 0.012	1.16 ± 0.21	24.18 ± 4.36	0.65 ± 0.086	5.18 ± 0.39	15.95 ± 0.16
Procyanidin B2	ND	ND	ND	ND	ND	ND	ND	ND	ND	ND	ND	ND	ND	ND	ND	ND
Epicatechin	1.462 ± 0.218	0.860 ± 0.089	0.063 ± 0.035	0.064 ± 0.007	1.02 ± 0.68	0.40 ± 0.01	0.78 ± 0.026	0.061 ± 0.002	0.23 ± 0.06	ND	0.11 ± 0.007	ND	1.045 ± 0.135	0.96 ± 0.12	0.123 ± 0.03	1.05 ± 0.069
Polydatin	ND	ND	ND	ND	ND	ND	ND	ND	ND	ND	ND	ND	ND	ND	ND	ND
Epicatechin gallate	ND	0.588 ± 0.016	ND	ND	ND	ND	0.082 ± 0.015	ND	ND	ND	ND	ND	ND	ND	ND	ND
Quercetin-3-galactoside	0.036 ± 0.0005	0.009 ± 0.0001	0.047 ± 0.008	ND	ND	ND	ND	ND	ND	ND	ND	ND	ND	ND	ND	ND
Quercetin-3-glucuronide	ND	0.006 ± 0.0001	0.014 ± 0.002	ND	ND	ND	ND	ND	ND	ND	0.036 ± 0.003	ND	ND	ND	ND	ND
Quercetin-3-glucoside	ND	ND	ND	ND	ND	ND	ND	ND	ND	ND	ND	ND	ND	ND	ND	ND
Kaempferol-3-glucoside	ND	ND	80.66 ± 2.33	ND	ND	ND	ND	ND	ND	ND	ND	ND	ND	ND	0.08 ± 0.003	ND
Quercetin-3-rhamnoside	ND	0.005 ± 0.0003	0.028 ± 0.003	ND	ND	ND	ND	ND	ND	ND	0.034 ± 0.004	ND	ND	ND	ND	ND
resveratrol	ND	0.15 ± 0.002	0.004 ± 0.001	ND	ND	0.008 ± 0.003	0.007 ± 0.001	ND	ND	ND	0.018 ± 0.004	ND	0.003 ± 0.001	ND	0.008 ± 0.0004	0.002 ± 0.0003
Quercetin	ND	0.007 ± 0.0002	ND	0.05 ± 0.008	ND	ND	0.007 ± 0.0003	ND	ND	ND	0.012 ± 0.001	ND	0.11 ± 0.016	0.01 ± 0.001	0.06 ± 0.004	0.03 ± 0.002
Kaempferol	0.003 ± 0.001	ND	ND	0.015 ± 0.01	ND	ND	ND	ND	ND	ND	ND	ND	ND	ND	0.01 ± 0.0	ND

*Data are expressed as mean ± standard deviation of triplicate experiments (*n* = 3) with confident interval significantly (*P* < 0.05).

*BA* Banda Aceh, *NS* North Sumatra, *WK* West Kalimantan, *WP* West Papua, *AN* Areca Nut, BQ Mix AN + betel leaf/betel inflorescence stem + slaked lime, *SI* stem inflorescence, *ND* Not Detected.

Flavanols, mainly catechin and epicatechin, were the most abundant polyphenols detected in nearly all samples except WK-leaf. Higher flavanols concentrations were observed in nuts compared to leaf, husk and SI. Epicatechin gallate was also detected in BA-Leaf at high concentrations, but not in other leaf samples. Flavonols, including quercetin-3-galactoside, quercetin-3-glucuronide, quercetin-3-rhamnoside, kaempferol-3-glucoside and quercetin were also detected in the leaf and husk samples mainly from BA, but low or absent in samples from NS, WK and WP. Resveratrol was the only stilbene detected in BQ mainly in leaf and husk, but absent or at low concentrations in nut samples.

### Alkaloid content in betel quid components

Only arecoline analytical standard showed a stable peak using the current method, while arecaidine, guvacine and guvacoline showed unstable peaks for standard curve. Therefore, arecaidine, guvacine, and guvacoline could not be identified and quantified with this method.

The highest arecoline concentration in the current study was observed in WP, followed by BA, WK, and NS respectively in a nut group alone (Table 3). Interestingly, in the mixture group, the concentration of arecoline in BQ from WP and WK dropped only 0.38 and 1.3 mg/g DM, respectively, compared to arecoline concentration in their AN. However, BQ mixtures from the other 2 regions showed a considerable reduction of arecoline concentration to more than 50% from their nut. No arecoline was detected in betel leaf and stem inflorescence samples. Low concentrations of arecoline was detected in husk from most regions, where husk from BA was the only husk which does not contain arecoline.

**Table 3:** Identification and quantification of arecoline by LC–MS analysis.

Samples	Arecoline (mg/g DM)
BA-AN	6.10 ± 0.99ab
BA-Leaf	ND
BA-Husk	ND
BA-Mix	2.58 ± 0.39d
NS-AN	4.11 ± 0.22c
NS-Leaf	ND
NS-Husk	0.37 ± 0.04 fg
NS-Mix	1.54 ± 0.23e
WK-AN	4.4 ± 0.27ac
WK-Leaf	ND
WK-Husk	0.16 ± 0.09
WK-Mix	3.11 ± 0.15d
WP-AN	**6.92 ± 1.64a**
WP-SI	ND
WP-Husk	1.01 ± 0.05ef
WP-Mix	5.57 ± 0.66b

*Data are expressed as mean ± standard deviation of triplicate experiments (*n* = 3) with confident interval significantly (*P* < 0.05). *ND* Not Detected. An *a* letter besides mean values refer to the highest value, and its value decreases as following the letter descending.

The bold value is significantly higher compared to another mean values.

The unbold value is significantly lower mean values.

### Association between BQ polyphenols and arecoline contents

**Figure 2:**
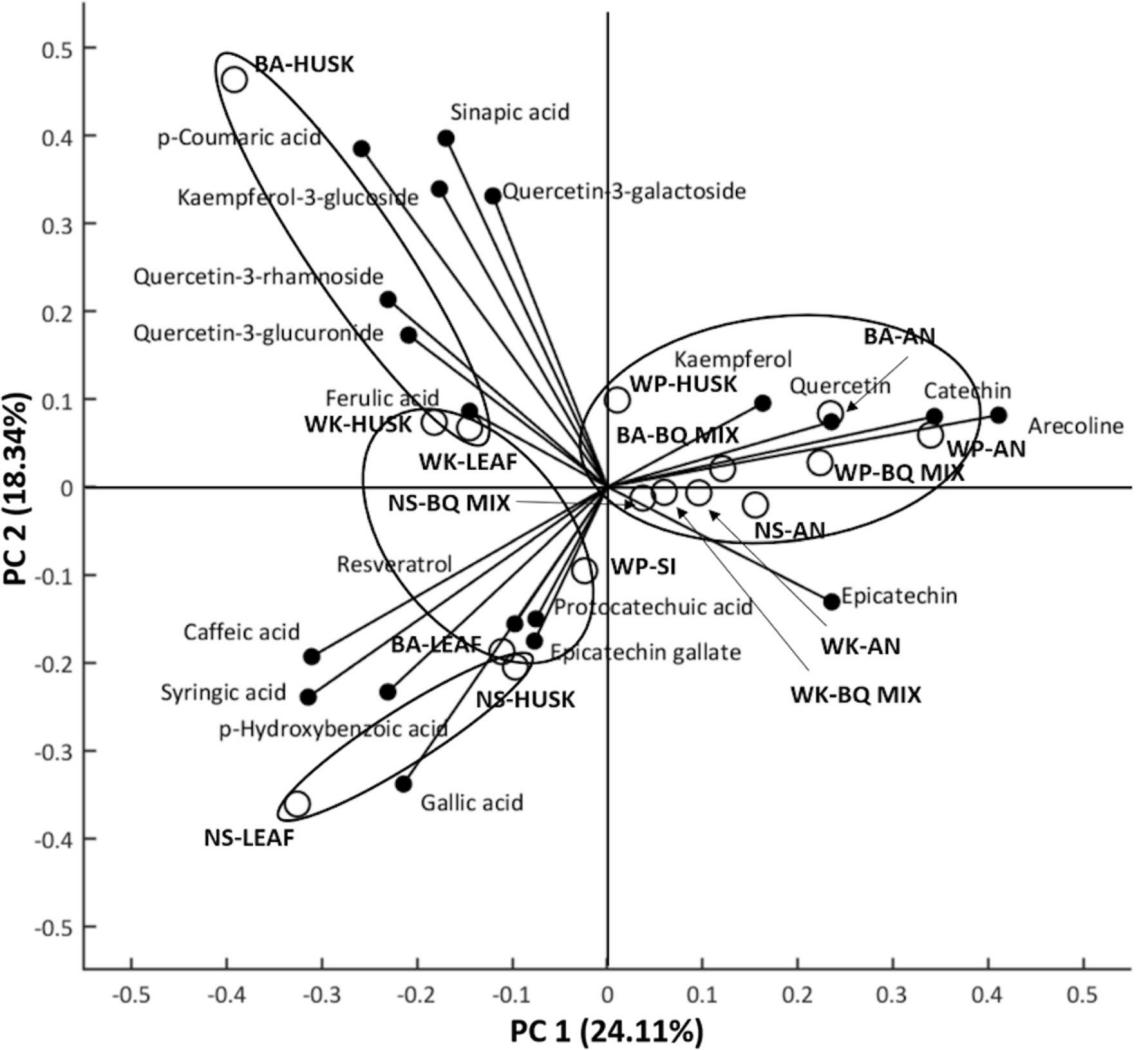
Principal component analysis (PCA) of betel quid phenolics and arecoline content among betel quid samples from 4 key regions of Indonesia. PC 1 explained 24.11% of total variance, while PC 2 explained 18.34% of total variance. *BA* = Banda Aceh, *NS* = North Sumatra, *WK* = West Kalimantan, *WP* = West Papua. *AN* = Areca Nut, *SI* = stem inflorescence *BQ Mix* = Areca nut + betel leaf/betel stem inflorescence. The samples on the positive aspect of PC 1 are mainly nuts and BQ mix samples with relatively high concentrations of catechin, epicatechin and arecoline. In this group, catechin and epicatechin have significantly positive correlation with arecoline. While samples on the negative aspect of PC 1 were similar for their low catechin, epicatechin and arecoline concentrations, but different in their concentration of other polyphenols. NS-leaf and NS-husk samples were well separated from others due to the abundant of phenolic acids. In this group, only *p*-hydroxybenzoic acid has significantly negative correlation with arecoline. While BA-leaf was distant from most samples due to its relatively high concentrations of flavonols.

Principal component analysis (PCA) was performed to systematically analyse the variations among different BQ samples in their phenolics and arecoline content (Fig. 2). Agglomerative hierarchical clustering (AHC) was further used to classify samples into different groups (Fig S3). A total of 42.45% of variation was explained by PC 1 (24.11%) and PC 2(18.34%). Arecoline, catechin, quercetin, kaempferol and epicatechin mainly contributed to positive aspect of PC 1, while sinapic acid, quercetin-3-galactoside, quercetin-3-rhamnoside, quercetin-3-glucuronide, sinapic acid, *p*-coumaric acid and ferulic acid contributed to negative aspect of PC 1 and positive aspect of PC 2. Protocatechuic acid, caffeic acid, syringic acid, *p*-hydroxybenzoic, gallic acid, epicatechin gallate and resveratrol contributed to the negative aspects of PC 1 and PC 2. Catechin, epicatechin on PC 1 were significantly positively correlated with arecoline, while *p*-hydroxybenzoic on the negative aspect of PC 2 was significantly negatively correlated with arecoline (Table 4).

**Table 4:** Correlation (Pearson) between arecoline and phenolic compounds.

Phenolics acid compounds	r-value	*P*-value
Ferulic acid	− 0.407	0.166
P-hydroxybenzoic acid	− **0.555**	**0.026**
Epicatechin	**0.515**	**0.041**
Syringic acid	− 0.481	0.059
Quercetin-3-rhamnoside	− 0.427	0.099
Kaempferol-3-glucoside	− 0.276	0.301
Catechin	**0.828**	** < 0.0001**
Quercetin	0.427	0.099
Caffeic acid	− 0.456	0.076
Gallic acid	− 0.300	0.259
P-coumaric acid	− 0.288	0.280
Epicatechin gallate	− 0.270	0.313
Quercetin-3-glucuronide	− 0.344	0.193
Quercetin-3-galactoside	0.024	0.931
Sinapic acid	− 0.080	0.767
Resveratrol	− 0.294	0.269
Kaempferol	0.312	0.239
Protocatechuic acid	− 0.2224	0.404

The bold phenolic acid compound is significantly correlated with arecoline.

The unbold phenolic acid compound is insignificantly correlated with arecoline.

Samples were well separated long PC 1 vectors (mainly arecoline, catechin and epicatechin) into two groups, while samples on the negative side of PC 1 would further be divided into three groups based on vectors of PC 2 (Fig. 2). The samples on the positive aspect of PC 1 are mainly nuts and BQ mix samples (WP-AN, WP-BQ mix, WK-AN, WK BQ-mix, BA-AN, BA-BQ mix, NS-AN) with relatively high concentrations of catechin, epicatechin and arecoline. In this group, catechin and epicatechin have significantly positive correlation with arecoline. While samples on the negative aspect of PC 1 were similar for their low catechin, epicatechin and arecoline concentrations, but different in their concentration of other polyphenols. NS-leaf and NS-husk samples were well separated from others due to the abundant of phenolic acids. In this group, only *p*-hydroxybenzoic acid has significantly negative correlation with arecoline. While BA-leaf was distant from most samples due to its relatively high concentrations of flavonols.

## Discussion

In this study we performed a systematic, thorough chemical characterisation of the individual constituents of BQ. Our data show, for the first time, that BQ has distinct chemical features that are mixture and region specific. Interestingly, the alkaloid and polyphenolic content as well as antioxidant activity of individual ingredients change significantly when these are combined into a BQ mixture. Different altitude and climate in each region may also account for the variation in polyphenols and arecoline content in the various BQ.

The results of the present study indicate that BQ from WP is of particular interest, and this is consistent with our previous epidemiology research^6^. Specifically, all forms of BQ from WP contain the highest concentrations of arecoline, phenolic acid, anthoxanthins, and stilbenes which may help explain the high prevalence of OSMF and oral cancer in this region. Further research is required to better elucidate the association between the identified arecoline and phenolics from four regions of Indonesia with the development of OSMF.

The highest arecoline concentration in the current study was observed in AN from WP, followed by AN from BA, WK, and NS respectively. The amount of arecoline changed significantly when AN was used in the BQ mixture but did so to a different extent. For example, the concentration of arecoline in WP-BQ mix and WK-BQ mix dropped only 1.35 and 1.3 mg/g DM respectively (WP-AN: 6.92 ± 1.64a, WP-mix: 5.57 ± 0.66b, WK-AN: 4.4 ± 0.27ac, WK-mix: 3.11 ± 0.15d). However, BQ mixtures from other 2 regions showed a considerable reduction of arecoline concentration to more than 50% from their nut with addition of slaked lime and leaf/SI (BA-AN: 6.10 ± 0.99ab, BA-mix: 2.58 ± 0.39d, NS-AN: 4.11 ± 0.22c, NS-mix: 1.54 ± 0.23e) (Table 3). These findings show that adding slaked lime not only decreases the overall concentration of polyphenols but, also, of arecoline. This might be due to extreme increases of pH up to 10 by slaked lime as most alkaloids are only stable in the range of pH 4–7^34^. This extreme pH has been shown to damage the alkaloid^35,36^, which might lead to decreased arecoline concentration. However, immature ANs from WK and WP were interestingly less affected by the addition of slaked lime, potentially due to different chemical and biological processes occurring in the mixture^37^. It could indicate that polyphenols and arecoline have a synergic protection from pH change that preserve the inclination number of arecoline i.e. the more phenolics in AN, the less arecoline damage by slaked lime in BQ mixture. However, further research is needed to clarify this hypothesis. One study from India reported that *Mangalore* variety of AN, extracted with various solvents, showed arecoline quantification of 2.3–12.79 mg/g DM^22,38^, whereas our current study using methanol extraction solvent showed a range of 0.37–6.9 mg/g DM (Table 3). This difference could be due to the origin of BQ products, different process or method of extraction, and its degree of maturity^39^. No arecoline was detected in betel leaf and SI samples. This finding was consistent with other studies, which reported high polyphenolic contents and absence of alkaloids in betel leaf and SI^26,40,41^. Low concentrations of arecoline was detected in husk from most regions, except BA-Husk. No previous study has investigated alkaloid and phenolics composition of husk, thus the present study is the first to assess the chemical composition of areca husk.

It has been suggested that regular and frequent polyphenols intake may be helpful to protect against oral cancer^19^. However, polyphenols can also act as pro-oxidant at high concentrations under certain conditions^14,15^. In the present study, we found polyphenols (mainly catechin and epicatechin) in high amount (Table 2), especially in WP-AN sample. Polyphenols induce cell apoptosis by triggering reactive oxygen species (ROS) through two mechanisms^13^, direct formation of labile aroxil radical or a labile radox complex with a metal cation^42^ and indirect activation of intracellular production of ROS by NADPH oxidases^43^. Cell culture study showed catechin-induced DNA damage when treating human leukemia HL-60 cells with epigallocatechin-3-gallate (EGCG) at 50 µM^16^. Further, in vivo studies show the dose-dependent hepatotoxicity of EGCG at single dose from 750–1500 mg/kg, where 1500 mg/kg treatment reduce survival rate by 85%^18^. Consistently, increasing numbers of hepatoxicity cases in humans have been associated with intake of green tea polyphenols, mainly EGCG^44^ and in some cases, associated with liver inflammation and necrosis^45^. Despite the results of a recent study that EGCG transiently inhibits both cell proliferation and migration of oral cavity cancer cells^46^, there is limited evidence showing the pro-oxidant effect of catechins and cytotoxicity to oral cells, which warrants further investigation.

Catechin as the main flavanol present in BQ can also act as antioxidant, that has been shown to prevent cell mutation by inhibiting the production of metalloproteases, leading to potentially reducing invasion and migration, inducing apoptosis and growth arrest in both oral cancer and oral leukoplakia cell lines^19^. The lower concentrations of catechin and other flavanols in mixture compared to nut observed in the current study may likely be due to the dilution effects of other BQ components. Furthermore, addition of lime can increase pH to 10 and damage the polyphenols^34^. Further, the polyphenols in AN have been proven to possess many pharmacological activities^47,48^. These include antiparasitic effects, anti-depressive effects, anti-fatigue effects, antioxidant effects, antibacterial and antifungal effects, antihypertensive effects, anti-inflammatory and analgesic effects, anti-allergic effects, the promotion of digestive functions, suppression of platelet aggregation, regulatory effects on blood glucose and lipids, etc.^20,47,48^. The large variations in BQ polyphenol contents of different Indonesian regions may be due to various reasons, such as environment conditions, germination, variety of AN, degree of ripeness^26,47^. It is to be noted that variations in the concentration of the various constituents may occur in nuts from different geographical locations and according to the degree of maturity of the nut. Similarly, the different altitude and climate in each region may play a role in the content of polyphenols and arecoline detected in our study, which makes it difficult to compare our results with those available in the literature.

In the current study, alkaloid and flavonoid (catechin and tannin) components were simultaneously identified in AN. The alkaloids are considered to be the most potent inducers of OSMF, while flavonoids have a synergistic role^7^. Flavonoids could decrease collagenase, stabilize the collagen fibrils to degradation by collagenase^8^.

Other polyphenols identified were ferulic acid and resveratrol that have been previously reported in AN^48,49^. However, in the present study, ferulic acid was not detected in any nuts samples and resveratrol was only detected in nuts from WP. Further polyphenols such as quercetin and kaempferol were also reported in AN^48,49^, and these compounds were detected in the current study and interestingly observed in leaf and husk samples as well. Previous studies on BQ polyphenols mainly reported phenolic compounds in the nuts^21,48–52^, but few study reported these compounds in husk, leaf and SI. Therefore, the polyphenols that were identified in husk, leaf and SI reported in the current study might explain the antioxidant capacity of these samples and provided insights for future research investigating antioxidants in BQ.

Sarode et al.^11^ hypothesized that only AN alone cannot trigger OSMF, without including other necessary required factors such as slaked lime and inflammation. These factors possibly result in conversion to phenotypically altered fibroblasts, which leads to increased fibrosis in the oral mucosa causing OSMF. Previous research has shown that powdered slaked lime applied to the chewed AN and betel inflorescence at the corner of the mouth in Papua New Guinea, causes the pH change to 10, at which reactive oxygen species (ROS) are produced from BQ in vitro^34^. This high pH change and excessive polyphenols can alter the polyphenols to act as pro-oxidant^13^. Thus, we hypothesise that high concentration of polyphenols in AN, especially catechin and epicatechin, together with high pH altered by slaked lime, are the main factors triggering OSMF. Further, our study showed that adding slaked lime into BQ mixture decreased by more than 70% all the antioxidant potency (TPC, FRAP and DPPH scavenging activities), compared to AN without slaked lime (Table 1). However, only WP-BQ mixture showed a slight inclination of polyphenols and arecoline concentration. In this case, consuming WP-BQ mixture results in having BQ with high concentration of polyphenols, arecoline and high pH due to added slaked lime. This could explain why people living in WP, who consume WP-BQ mixture, are more likely to develop OSMF as shown in our previous study^6^. Further in vitro and in vivo research needs to be conducted to elucidate the association between Indonesian BQ and pathogenesis of OSMF.

The observed high TPC value of WP-AN compared to AN from other regions could reflect the different amount of immature nuts from this region, as unripe ANs commonly contain more polyphenols compared to ripe AN^26^. This theory is consistent with our current study that the unripe AN from WP and WK showed the highest total phenolics content. Further, we also found that FRAP and DPPH antioxidant activities are higher in unripe nut rather than in the ripe AN from BA and NS (Table 1). The TPC value of nut may also depend on the region where areca catechu is grown, and its processing method^39^. In present study, we could recognize unripe AN by its green in colour, while the ripe AN looks yellow (Fig. 1)^26^. In some countries, such as Taiwan, Guam, and China, the unripe AN is mostly consumed^26^, similar to WP and WK. Whereas in BA and NS, the ripe AN is favoured^6^.

An interesting finding was that the phenolic content and antioxidant activities dropped in the BQ mixture featuring slaked lime (AN: betel leaf/SI: slaked lime = 80.5:12.5:7 by weight). A previous study also reported that BQ without slaked lime had higher TPC, antioxidant and cytoprotective activities compared to BQ with slaked lime^22^. It may be that slaked lime raises the pH up to 10 leading to phenolics damage^34^. TPC in BQ mixture from BA, NS, and WK dropped by 94.5%, 90.5%, and 74.5% compared to TPC of AN alone, respectively. These results were consistent with FRAP and DPPH tests as well (Table 1). Interestingly, total phenolics content in WP-BQ mixture only dropped by 28.4% from the amount in AN alone. This might be due to the degree of immaturity of WP-AN and the form of slaked lime. The excessive amount of phenolics could lead to cytotoxicity instead of exerting protective action^19^. The results of our recent survey supports the theory that consuming excessive polyphenols in BQ could be detrimental to, rather than protective for oral health^19^. Our previous study showed that 60 out of 139 participants (43.17%) who had OPMD were living in WP region and mostly BQ consumers^6^. This correlates in this current study with BQ containing the most abundant phenolics levels. Thus, we suggest consuming BQ containing ripe AN is better compared to consuming BQ containing unripe AN, as ripe AN contains less arecoline, known to trigger OSMF development^8^, and contains moderate number of polyphenols that can act to protect oral cavity^19^.

WK-leaf had the highest antioxidant activities and the widest range of phenolic acids among betel leaf/stem group alone. WP-SI was observed to have the highest concentration of phenolic compounds, such as *p*-hydroxybenzoic acid. Previous study reported that betel SI may contain polyphenols such as hydroxychavicol, eugenol, isoeugenol, eugenol methyl ester and safrole^40^. Safrole as the major polyphenols in betel inflorescence is also considered to be a carcinogen^41^. However, safrole was not profiled in the present study. The values of FRAP and DPPH were consistent with TPC results in most samples, indicating that FRAP and DPPH attributes of the samples were due to the presence of polyphenol compounds.

Some people living in Taiwan, China, Guam, and Papua New Guinea include the husk of outer AN pericarp as part of the BQ mixture^26^. This might increase the risk of developing OSMF, as our study revealed that husk from WK, NS, and WP contains 0.16, 0.37, 1.1 mg/g DM of arecoline respectively (Table 3). Further, an improper management of waste of husk could also possess potential hazardous to environment as it contains carcinogenic properties. The recent study suggested the use of areca husk as self-assembly of oleylamine modified nano-fibrillated cellulose from areca husk fibers into giant vesicles formed that can have applications in storage and delivery of drugs in topical applications^53^. Thus, our findings describing the chemical composition of areca husk is important to avoid or manage future risks. Further study is required to clarify how husk can hazardous the environment. There betel husk should be carefully managed to avoid entering human food chain.

## Conclusion

BQ compounds have distinct chemical features that are mixture and region specific. Interestingly, the alkaloid and phenolic content as well as antioxidant activity of individual ingredients change significantly when these are combined into a BQ mixture. We observed that BQ compounds from WP was distinctive from other regions, and that AN and BQ mixture from this region contained the highest TPC, antioxidant activities, polyphenols and arecoline content.

Catechin and epicatechin are the main polyphenols detected in BQ, while WP had the highest concentrations in nut sample. The high amount of catechin and epicatechin could change the effect of antioxidant into pro-oxidant leading to raise the potential of developing OSMF.

Arecoline was detected in all ANs and mixture samples and was significantly correlated with catechin and epicatechin, and significantly negatively correlated with *p*-hydroxybenzoic acid. Phenolic acids and flavanols content in betel quid mixture were low, likely due to the addition of lime, which also led to lower antioxidant activity and radical scavenging capacity in the mixture.

The ripeness degree of AN is directly related to the amount of both polyphenols and arecoline. The unripe AN contained a higher concentration of polyphenols and arecoline compared to ripe AN. Suggesting that consume the ripe AN could lower the potential in developing OSMF.

In summary, our results shed light on the chemical composition of different Indonesian BQs and may inform the development of chemo-preventive strategies to contrast the development of OSMF. For example, consuming mature type AN, avoiding the husk because of its high arecoline content, as well as not adding slaked lime, could decrease the potential of developing OSMF in betel chewers”.

## Supplementary information

Supplementary file1.
