# Supplementary material for: Distinct phenolic, alkaloid and antioxidant profile in betel quids from four regions of Indonesia
Source: Sci Rep. 2020 Oct 1;10:16254. doi: 10.1038/s41598-020-73337-0 (PMC7529777; doi:10.1038/s41598-020-73337-0)
Supplement: Supplementary file 1 — Supplementary file1. [file 41598_2020_73337_MOESM1_ESM.docx]

**Characterization of phenolics and arecoline constituent profile in betel quids from four regions of Indonesia**

Authors: *Elizabeth Fitriana Sari^1,3,4^, Grace Puspita Prayogo^2^, Yit Tao Loo^2^, *Pangzhen Zhang^2^, Michael John McCullough^1^, and *Nicola Cirillo^1^

*^1^Melbourne Dental School, The University of Melbourne, 720 Swanston Street, Carlton, VIC,*

*3053, Australia*

*^2^School of Agriculture and Food, Faculty of Veterinary and Agricultural Sciences, The*

*University of Melbourne, Parkville, VIC, 3052, Australia*

*^3^Faculty of Dentistry, Universitas Padjadjaran, Jl. Raya Sumedang KM 21, Jatinangor,*

*45363, Indonesia*

*^4^Dentistry and Oral Health, La Trobe Rural Health School, La Trobe University, Bendigo,*

*VIC, 3550, Australia*


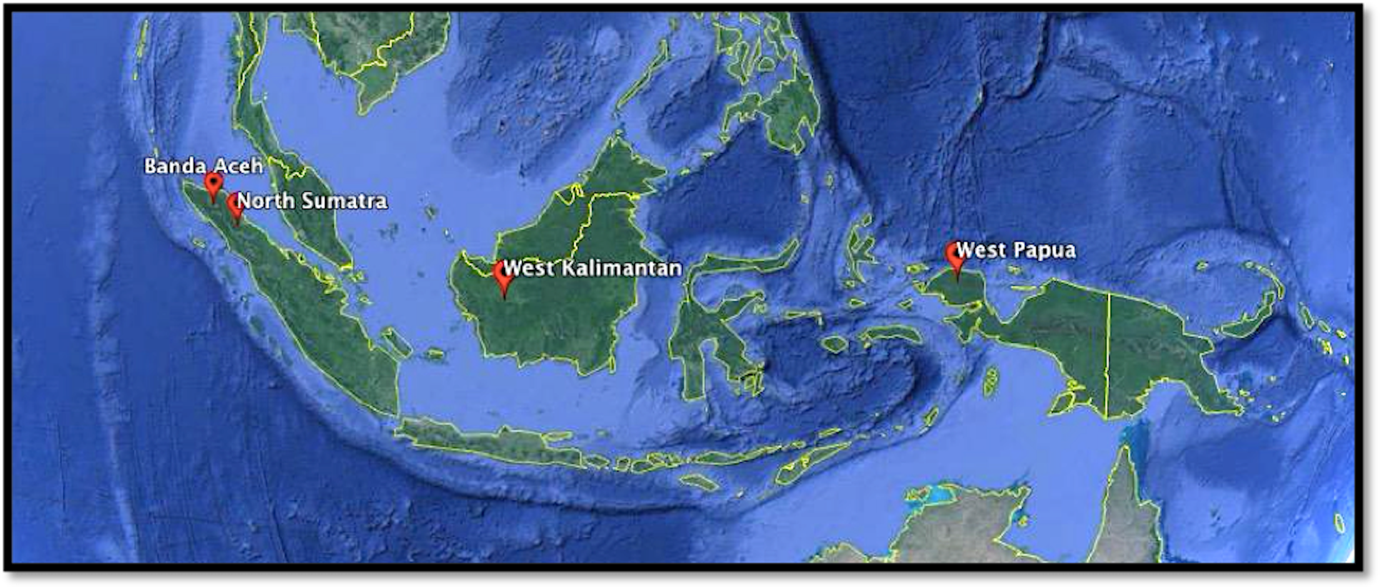


**Fig S1.** Betel Quid imported from four key regions of Indonesia which were Banda Aceh (BA), North Sumatra (NS), West Kalimantan (WK) and West Papua (WP). Map data; Google, Image Landsat/ Copernicus).

**
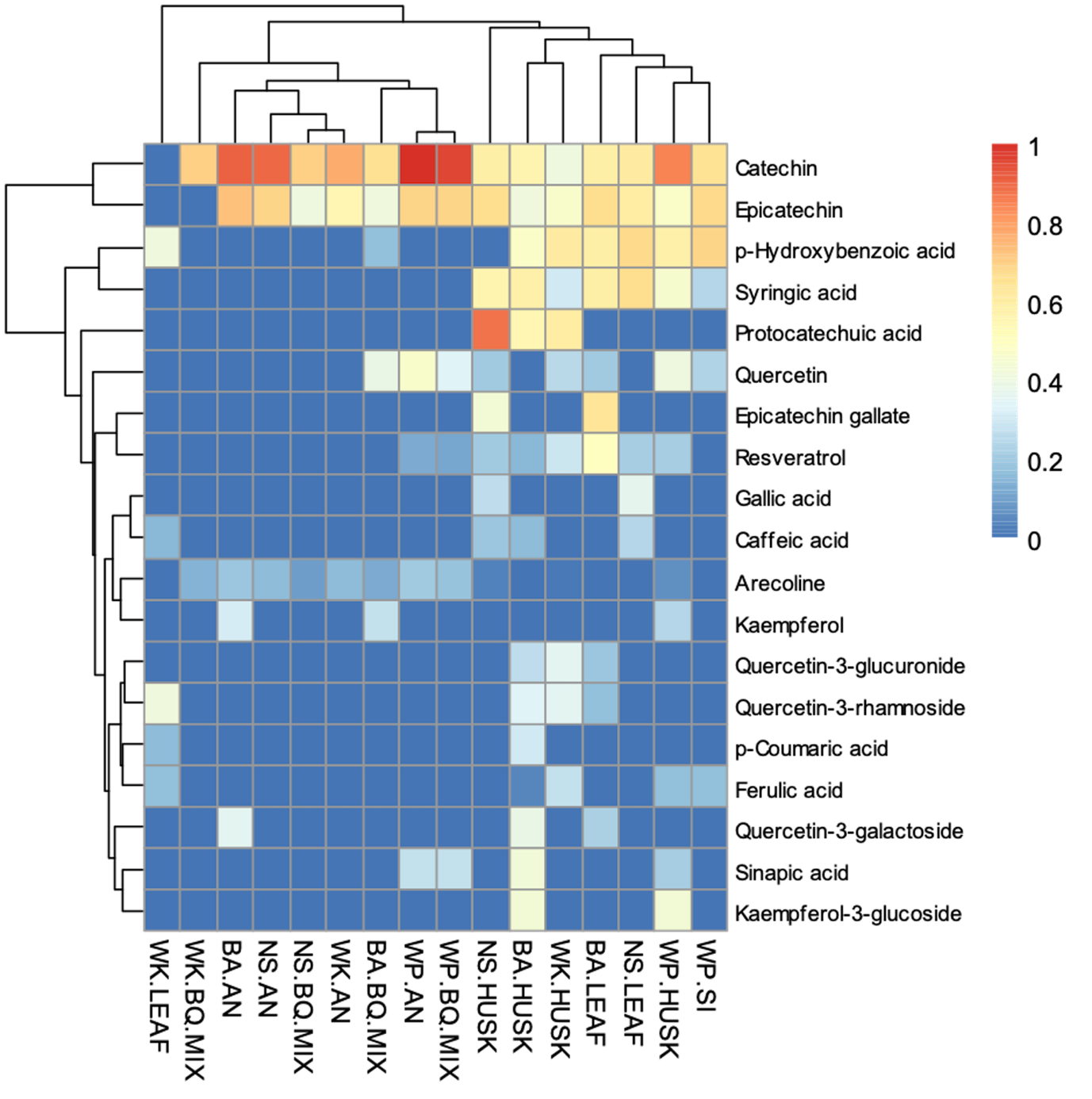
**

**Fig S2**. Heat Map showing arecoline and polyphenols that were identified using LC-MS in the samples.

BA = Banda Aceh, NS = North Sumatra, WK = West Kalimantan, WP = West Papua.

AN= Areca Nut, SI= stem inflorescence *BQ Mix* = Areca nut + betel leaf/betel stem inflorescence. Respectively, red colour in gradient to blue colour in the heat map shows the highest, moderate, and lowest concentration of identified polyphenols and arecoline.


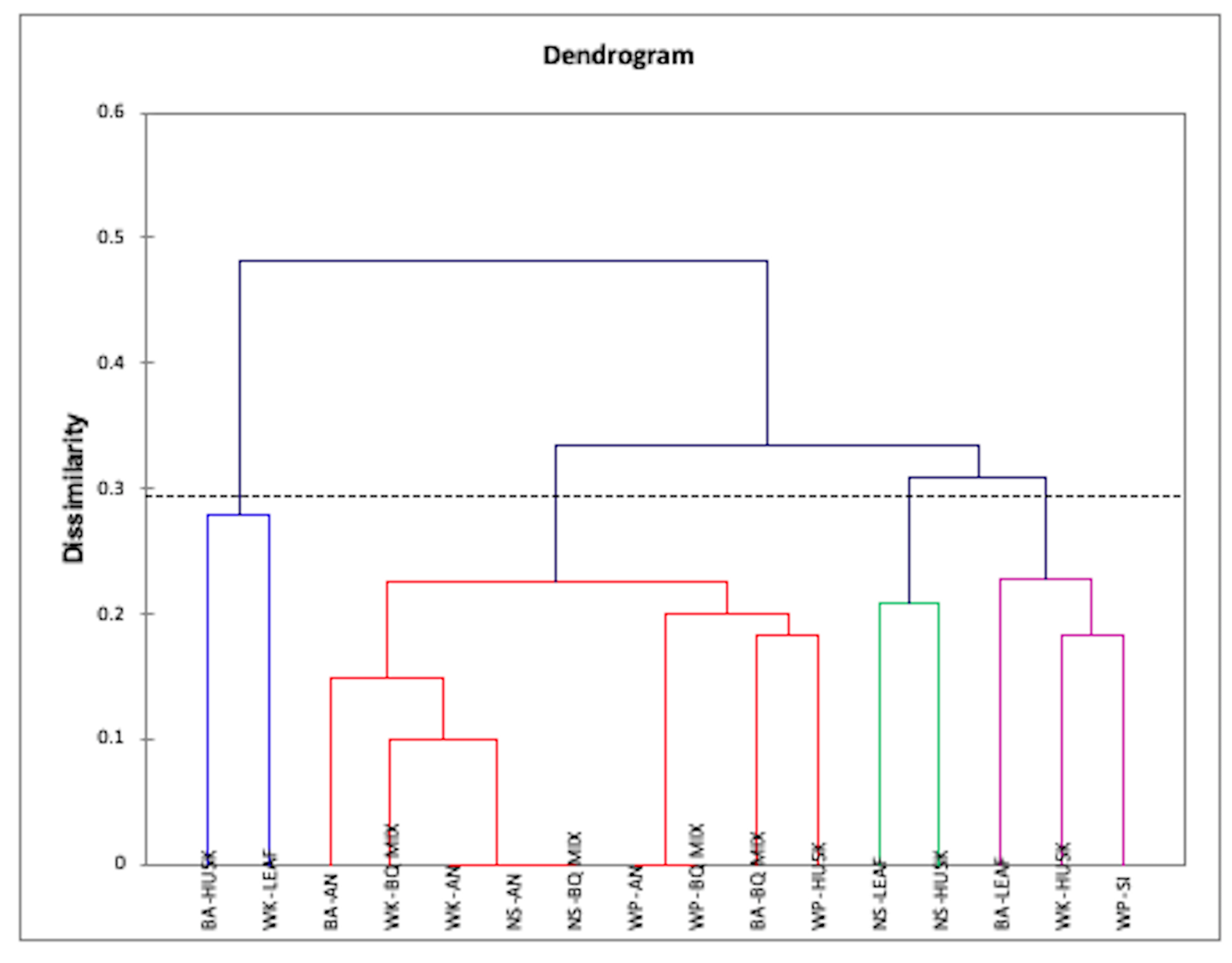


**Fig S3.** Agglomerative hierarchical clustering analysis for the betel quid samples based on polyphenols and arecoline content that were identified using LC-MS.

BA = Banda Aceh, NS = North Sumatra, WK = West Kalimantan, WP = West Papua.

AN= Areca Nut, SI= stem inflorescence *BQ Mix* = Areca nut + betel leaf/betel stem inflorescence

**Table S1.** LC-MS Data of Phenolic Compounds Extracted from Various BQ from Indonesia

| **Name of Compound** | **Retention Time (minute)** | **Fragment m/z** |
| --- | --- | --- |
| ***Hydrocinamic Acid*** | | |
| Ferulic Acid | 37.005 | **193**, 134 |
| Caffeic Acid | 25.881 | **179**, 135 |
| P-coumaric Acid | 33.518 | **163**, 119 |
| ***Hydrobenzoic acid*** | | |
| Gallic Acid | 7.187 | **169**, 125 |
| Protocatechuic Acid | 12.459 | **153**, 109 |
| p-hydroxybenzoic Acid | 19.92 | **137**, 93 |
| ***Other Phenolic acids*** | | |
| Trans - sinapic acid | 38.006 | **223**, 164 |
| Syringic Acid | 26.881 | **197**, 153/182 |
| Caftaric Acid | 13 | **311**, 179 |
| 5-hydroxymethyl furfural | 2.064 |  |
| Polydatin | 38.101 | **389**, 227 |
| ***Stilbense*** | | |
| Resveratrol | 52.888 | **227**, 185 |
| ***Falvanols (Flavanoids group)*** | | |
| Catechin | 22.353 | **289**, 245 |
| Epicatechin | 28.206 | **289**, 245 |
| Epicatechin gallate | 37.117 | **441**, 289 |
| ***Flavonols (Flavanoids group)*** | | |
| Quercetin | 64.985 | **301**, 151 |
| Quercetin 3-o galactoside | 40.611 | **463**, 301 |
| Quercetin 3-o glucuronide | 46.81 | **477**, 301 |
| Quercetin 3-o rhaminoside | 47.311 | **447**, 301 |
| Kaempferol | 66.586 | **285**, 249 |
| Kaempferol 3-o glucoside | 46.86 | **447**, 284/285 |
| Quercetin 3-O Glucoside | 41.633 | **463.2**, 301 |
